# Automating clinical phenotyping using natural language processing

**DOI:** 10.1038/s43856-025-01337-0

**Published:** 2026-01-14

**Authors:** Linea Schmidt, Susanne Ibing, Florian Borchert, Julian Hugo, Allison A. Marshall, Jellyana Peraza, Judy H. Cho, Erwin P. Böttinger, Bernhard Y. Renard, Ryan C. Ungaro

**Affiliations:** 1 Hasso Plattner Institute, Digital Engineering Faculty, University of Potsdam, Potsdam, Germany; 2 Hasso Plattner Institute for Digital Health at Mount Sinai, Icahn School of Medicine at Mount Sinai, New York, NY USA; 3 Windreich Dept. of Artificial Intelligence & Human Health, Icahn School of Medicine at Mount Sinai, New York, NY USA; 4 Department of Medicine, Mount Sinai Health System, New York, NY USA; 5 The Henry D. Janowitz Division of Gastroenterology, Icahn School of Medicine at Mount Sinai, New York, NY USA; 6 Department of Pathology, Molecular, and Cell Based Medicine, Icahn School of Medicine at Mount Sinai, New York, NY USA

**Keywords:** Crohn's disease, Medical research

## Abstract

**Background:**

Real-world studies based on electronic health records often require manual chart review to derive patients’ clinical phenotypes, a labor-intensive task with limited scalability. Here, we developed and compared computable phenotyping based on rules using the spaCy framework and a Large Language Model (LLM), GPT-4, for sub-phenotyping of patients with Crohn’s disease, considering age at diagnosis and disease behavior.

**Methods:**

For our rule-based approach, we leveraged the spaCy framework and for the LLM-based approach, we used the GPT-4 model. The underlying data included 49,572 clinical notes and 2204 radiology reports from 584 Crohn’s disease patients. A test set of 280 clinical texts was labeled at sentence-level, in addition to patient-level ground truth data. The algorithms were evaluated based on their recall, precision, specificity values, and F1 scores.

**Results:**

Overall, we observe similar or better performance using GPT-4 compared to the rules. On a note-level, the F1 score is at least 0.90 for disease behavior and 0.82 for age at diagnosis, and on patient level at least 0.66 for disease behavior and 0.71 for age at diagnosis.

**Conclusions:**

To our knowledge, this is the first study to explore computable phenotyping algorithms based on clinical narrative text for these complex tasks, where prior inter-annotator agreements ranged from 0.54 to 0.98. There is no statistical evidence for a difference to the performance of human experts on this task. Our findings underline the potential of LLMs for computable phenotyping and may support large-scale cohort analyses from electronic health records and streamline chart review processes in the future.

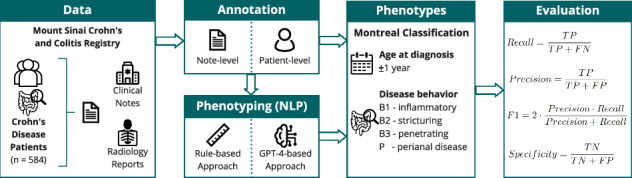

## Plain language summary

Doctors and researchers often need to group patients by specific medical features (called “phenotypes”) to study the disease and improve care. Much of this information is in free-text clinical notes rather than in tabular data. As an example, for patients with Crohn’s disease, the free-text notes can include important details describing the disease course over time, such as bowel narrowings (strictures), abnormal openings (fistulas), problems with the area around the anus, and age at diagnosis. Prior studies show that relying only on structured data, such as codes used to describe particular diseases, often miss these types of details. Reading notes by hand is more accurate but slow and costly. Natural language processing (NLP) is a computational method to automatically read and extract this information from clinical text. We built two NLP approaches and created new sentence-level datasets to test them. Both approaches found complications well, with combining both approaches giving the most balanced results. This method could save time in research and help clinicians flag people who may need extra care.

## Abstract (web summary)

Schmidt et al. develop sentence-level datasets for Crohn’s phenotypes and compare rule-based NLP with GPT-4 to extract disease behavior and age at diagnosis from EHR notes. Both methods achieve high recall on notes; GPT-4 perfectly identifies age at diagnosis and simple ensembles improve precision and enable chart-review prioritization.

## Introduction

Computable clinical phenotyping, the automatic grouping of patients according to their medical history captured in Electronic Health Records (EHR), has been the focus of numerous studies in the past decades^1,2^. In these approaches, manual chart review is limited to developing and validating the phenotyping algorithms using a labeled subset, rather than annotating entire datasets—thereby enabling scalable clinical research. Large consortia such as Electronic Medical Records and Genomics (eMERGE) have invested in resources such as the Phenotype Knowledge Base, which focuses on developing, sharing, and validating computable phenotyping algorithms^3^.

These algorithms can broadly be divided into two main categories: rule-based and machine learning (ML)-based approaches. The choice between these approaches relies on factors like the availability of data and the complexity of the task at hand^4^. Rule-based techniques utilize predefined rules or criteria, typically requiring expertise in the relevant medical domain. In contrast, ML-based methods employ algorithms to recognize data patterns corresponding to different phenotypes.

Often, phenotypes of patient subgroups are insufficiently captured in the structured EHR, requiring analysis of clinical narrative text and the use of natural language processing (NLP) to derive these phenotypes^5^. In their benchmark paper, Moldwin et al. demonstrated, among others, for digestive diseases, that the incorporation of unstructured data outperforms models that are only based on structured EHR^6^. While ML-based approaches may be able to identify unknown patterns using vast amounts of data, a rule-based approach provides increased transparency compared to ML approaches, particularly when applying large language models (LLMs).

As Ananthakrishnan et al. demonstrated, one example of phenotypes that cannot be reliably extracted from solely structured EHR are clinical subgroups of Crohn’s Disease (CD)^7^. CD, one of the main types of inflammatory bowel disease (IBD), is an immune-mediated disease marked by recurrent episodes of chronic inflammation of the gastrointestinal (GI) tract. The disease is characterized by significant heterogeneity in disease course and treatment response^8^. In a recent publication, an expert consensus of the European Crohn’s and Colitis Organization (ECCO) discussed core outcomes and outcome measures relevant to be reported in IBD studies based on real-world data, such as EHR^9^. When reporting or studying disease complications, they recommended considering the disease phenotype according to the Montreal Classification, such as the presence of strictures or fistulas, as core outcomes (Table A.1)^10,11^.

**Fig. 1:**
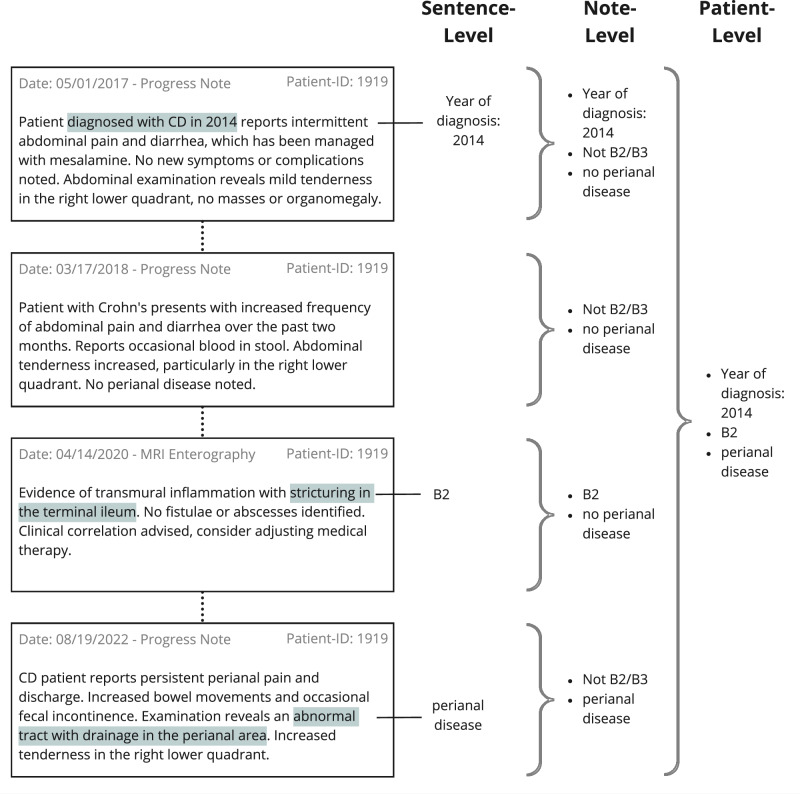
Sentence-, note-, and patient-level labeling for an example patient. Four different fictional notes demonstrate the information extraction flow on sentence-, note-, and patient-level. Higher-level information requires aggregation of multiple data points.

Disease behavior comprises different disease complications of CD, for instance, strictures (B2) or fistulas and abscesses (B3). Strictures are luminal narrowings in any part of the GI tract, derived from a mixture of inflamed and scarred tissue^12^. Fistulae are abnormal connections between different parts of the GI tract, between the GI tract and other organs, or between the GI tract and the skin^8,13^. An abscess is a localized accumulation of pus that can develop due to inflammation or infection^14^. Perianal disease is regarded as a disease modifier that can co-occur with any other disease phenotype (B1-B3): Any disease complication in the perianal region is considered perianal disease. Disease behavior is not a static classification category but changes during the course of the disease^15^. The nuanced and often complex descriptions of the behavioral phenotype in clinical text include descriptions of symptoms and treatment responses, as well as the progression of the disease (Fig. 1). Since phenotype data are stored mainly in clinical narrative text, a patient’s disease behavior is usually extracted by manual chart review^16–18^.

Age at diagnosis, another category of the Montreal Classification, refers to the age at initial CD diagnosis, and disease location to the intestinal region of inflammation. Next to disease behavior, age at diagnosis is an essential clinical component for CD clinical care and study cohort characterization, as it allows the deduction of disease duration, a prognostic factor for treatment response with biologics^19^.

Automated phenotyping based on NLP techniques, including information from clinical notes, could facilitate patient classification on a large scale with minimal manual labeling required. For instance, Stidham et al. recently demonstrated the successful extraction of extraintestinal manifestations in IBD patients using a rule-based NLP approach^20^.

For CD, a study on structured EHR data from the Swedish National Patient Register (*n *= 1403) analyzed whether ICD codes accurately represent IBD subgroups and phenotypes according to the Montreal classification. The study compared the ICD-coded information with available data from EHR, utilizing the results from patient-level chart review as a reference, excluding patients without the corresponding information. As a result, recall values of 75% for B1, 62% for B2/B3 and 81% for perianal disease were reported^21^, highlighting the need for more granular datasets and algorithms leveraging unstructured EHR data. Most existing datasets are based on chart reviews and only include higher-level annotations on the patient level^22–24^.

In this work, we describe the development of two sentence-based labeled datasets, including annotations of disease phenotype and age at diagnosis in clinical notes of CD patients. We use these data to develop and evaluate rule-based phenotyping algorithms and compare them with an in-context learning approach using a GPT-4 model from OpenAI. The GPT-4 model performs similarly or better than the carefully curated rules. The established pipeline facilitates the large-scale labeling of previously unannotated clinical narrative text and demonstrates the applicability of a large language model (LLM) in this context.

## Methods

### Data collection and preprocessing

Clinical notes from the EHR of the Mount Sinai DataWarehouse (MSDW) between February 1940 to May 2023 were acquired via the Artificial Intelligence Ready Mount Sinai (AIR ⋅ MS) platform^25^. The study was approved by the Program of Protection for Human Subjects at the Icahn School of Medicine at Mount Sinai (21-01782). This dataset was further enriched with computed tomography (CT) and magnetic resonance imaging (MRI) reports from the radiology department. In total, 792 CD patients from the Mount Sinai Crohn’s and Colitis Registry (MSCCR) were considered for this study due to already existing annotations on a patient-level^16^. All participants provided written informed consent prior to enrollment in the study. We preprocessed the available clinical notes and radiology reports by removing irrelevant note types (e.g., telephone encounters and patient instructions) and texts without CD-related context (Fig. A.1). Of the 792 patients, 584 had at least one non-empty clinical note available after filtering. For the annotation and extraction of the age at diagnosis, we additionally filtered notes for regular expressions containing key expressions such as “diagnosed”, “Crohn’s […] since”, or “age at” (Section A.3.1). To allow for further granularity, we split all clinical texts into sentences (Fig. A.1).

### Annotation and dataset creation

For disease behavior, annotation guidelines were based on Montreal classification annotation guidelines from the COMPASS-IBD study and the Ocean State Crohn’s and Colitis Area Registry (OSCCAR)^17,18^. The annotation is based on two different categories: disease behavior (B1/B2/B3) and perianal disease (yes/no). Two annotators, an internal medicine resident, and an IBD researcher, labeled overall 200 notes, in total 15,390 sentences, on a sentence-level (Fig. A.2). This includes an agreement sample of 50 notes (5543 sentences), which was used to ascertain the Inter-Annotator Agreement (IAA), measured using Cohen’s kappa statistics^26^. After resolving disagreements on the IAA sample, a curated dataset was created and used as a test set containing 200 notes. We additionally labeled a development set consisting of 200 clinical texts, labeled by non-experts. Rules and prompts were exclusively developed using this development dataset and evaluated on previously unseen test data. To allow an evaluation of the disease behavior on the patient level, we used a labeled subset of 134 patients from MSCCR.

The annotation of the age at diagnosis was conducted separately from the disease behavior by the IBD researcher, labeling a different sample of 200 clinical texts on a sentence level based on three categories: age at diagnosis, diagnosis year, and disease duration. Notably, the ground-truth age at diagnosis was calculated using patients’ birth years and the note dates. After curation, this dataset was split into a testing and a development set. The original labels from MSCCR patients with available clinical texts were used for a patient-level evaluation.

### Rule-based phenotyping

#### Disease behavior

**Fig. 2:**
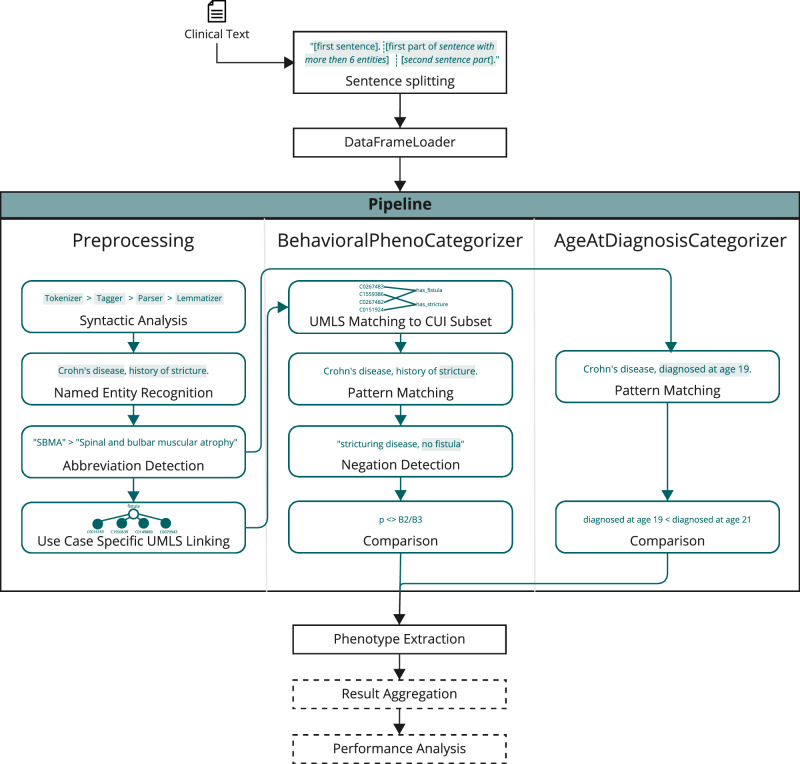
Rule-based phenotyping algorithm. The phenotyping pipeline includes multiple preprocessing steps, as well as the newly developed BehavioralPhenoCategorizer and the AgeAtDiagnosisCategorizer. After the phenotype extraction, result aggregation and performance analysis are optional. Evaluation is only conducted if ground-truth labels are available.

The rule-based approach leveraged spaCy for syntactic analysis and pattern matching^27^, scispaCy for concept extraction using the *en_core_sci_md* model^28^, and medspaCy for negation detection^29^. In brief, following preprocessing, including sentence splitting for sentence-level analysis, abbreviation detection, and Unified Medical Language System (UMLS) linking, a custom spaCy component, BehavioralPhenoCategorizer, was used to match multiple patterns (Fig. 2). For negation detection, in addition to medSpacy, a Transformer-based Clinical Assertion and Negation Classification BERT model was used^30^. Phenotypes were aggregated at different levels (patient, note, or sentence), with the most severe phenotype following the order B1 < B2 < B3, and stored together with the notes’ timestamps. More details can be found in Section A.3.2.

#### Age at diagnosis

Similar to the disease behavior, a custom spaCy component, AgeAtDiagnosisClassifier, was engineered to determine the age at diagnosis. Through a series of pattern matching, age at diagnosis was extracted based on three types of expressions in the narrative text: (1) directly stated age, (2) year of diagnosis, and (3) disease duration. When only the year of diagnosis was given, age was computed by subtracting it from the year of MSCCR enrollment (patient-level) or the note/report year (note-level). For disease duration, values given in months were converted to years and subtracted from the patient’s age at enrollment or note creation, respectively. Of note, the identified year of birth includes an error margin of ±1 year since exact dates are not frequently mentioned in clinical notes. In cases where differing ages were extracted from various notes, we selected the earliest age at diagnosis during patient-level aggregation.

### In-context learning

We deployed the Azure OpenAI GPT-4 model (1106-Preview) to generate note-level structured output on disease behavior phenotypes^31^. For the prompt, we added the slightly shortened annotation guidelines of the COMPASS-IBD study and three randomly selected examples, including outputs from the development dataset, an approach considered as few-shot prompting (Section A.3.3). The model was used off-the-shelf without any fine-tuning. We implemented a function call to pre-define and structure the output of the GPT-4 model. To make the model results more deterministic, we set the temperature parameter of the model to 0. Besides that, the OpenAI default parameters were used.

### Algorithm performance evaluation

The phenotyping algorithms were evaluated on note- and patient-level using the annotated test datasets. The evaluation per class was conducted based on recall, precision, specificity, and F1 score. Per class evaluation was chosen to account for class imbalance and ensure comparability with prior literature. During the development of the disease behavior rule-based phenotyping algorithm using the separate development dataset, our primary metric of interest was recall, given the importance of the sensitive identification of positive instances. For age at diagnosis, we prioritized precision to focus on the accurate extraction of the information for downstream tasks. Here, True Positives (TP) corresponded to accurately identified ages at diagnosis (within ±1-year) for performance metrics. True negatives (TN) were accurate identifications where age information was absent, false positives (FP) were defined as wrongly identified age at diagnosis labels, and false negatives (FN) represented overlooked labeled instances. Maintaining a balanced precision, F1 score, and specificity were set as secondary aims.

## Results

### Curation of the disease behavior and age at diagnosis datasets

The quality of the disease behavior labeling process is determined through Cohen’s kappa agreement scores on the 50 notes that are labeled by both annotators. On sentence-level, we observe an overall IAA score of 0.85 (Not B2/B3: 0.83; B2/B3: 0.84; perianal disease: 0.87; Table A.3). Evaluation on note-level increases the overall IAA to 0.90. These results indicate a good consensus among annotators^32^.

After the annotators reached a consensus for all disagreement instances, in the finalized, curated test set, approximately 1% of clinical note sentences and 3.6% of radiology report sentences are labeled as B2 or B3 (Table A.2). In total, 0.8% of clinical note sentences and 2.1% of radiology report sentences are annotated with perianal disease.

For age at diagnosis, we label a total of 80 randomly selected clinical texts and a total of 12,293 sentences (Table A.4). Most commonly, the year of diagnosis is stated (60%), followed by the age of diagnosis (23%) and disease duration (10 %).

For patient-level annotations, the complete patient chart is used up until the MSCCR study enrollment. All 584 patients are labeled for age at diagnosis, and a subset of 134 patients for disease behavior.

### Effective disease behavior extraction on note-level with rules and GPT-4

We use full note texts for GPT-4 input, but run the rule-based approach at the sentence level, aggregating results for note- or patient-level evaluation. To optimize our spaCy pipeline, we experiment with varied settings regarding rule types and negation detection options on the development set. A synergistic approach combining UMLS matching rules with rules for direct string matching is superior in its performance compared to applying either of the rule types alone. Notably, the exclusive employment of string matching exhibits superior results compared to relying solely on UMLS matching across clinical notes and radiology reports (Fig. A.3). For negation detection, we analyze the number of false positives and negative disease complications using the medspaCy negation detection component, the LLM Negation Classifier, or no negation detection. The LLM Negation Classifier performs superior to the other two options, manifesting the fewest false negatives while preserving a substantial number of accurately identified instances (Figs. A.4 and A.5).

Using either the rule-based or LLM-based approach, the automated behavioral phenotyping based on clinical notes yields high recall values on note-level, ranging from 0.92 to 1.00, depending on the phenotype (Table 1). The identification of no existing disease complications is particularly successful, with recall and precision values above 0.94 for both the GPT-4 and rule-based approach. Overall, the two approaches perform very similarly at the note-level. Also, when extracting the disease complication categories individually, we are able to identify all instances of B3 (Table A.5). Nonetheless, we improve the results by aggregating B2 and B3 into one joint disease complication category compared to separately phenotyping B2 and B3. A more detailed error analysis of both approaches is provided in Tables A.9 and A.10. The calculated Cohen’s kappa agreement scores between the labels from the annotated consensus dataset and the labels derived from the rules or GPT-4 are on average between 0.83 and 0.86 (Table A.3). These scores are comparable and statistically not inferior to the IAA between the human annotators, as confirmed by Z-tests showing no significant difference (all *q*-values ≥ 0.70; Table A.6). This underlines the high performance of the computational approaches.

**Table 1:** Performance of the rule-based phenotyping algorithms and in-context learning using a GPT-4 model to extract disease behavior on note-level using the newly annotated test datasets comprised of 150 clinical notes

Phenotype	Model	Recall	Precision	F1 score	Specificity
Not B2/B3	Rules	0.94	1.00	0.97	0.84
	GPT-4	0.95	0.98	0.96	0.86
	Rules AND GPT-4	0.98	1.00	0.99	0.94
	Rule OR GPT-4	0.90	1.00	0.95	0.78
B2/B3	Rules	1.00	0.84	0.92	1.00
	GPT-4	0.95	0.86	0.90	0.98
	Rules AND GPT-4	1.00	0.94	0.97	1.00
	Rule OR GPT-4	1.00	0.78	0.87	1.00
Perianal—yes	Rules	1.00	0.86	0.93	0.86
	GPT-4	0.92	0.92	0.92	0.92
	Rules AND GPT-4	1.00	1.00	1.00	1.00
	Rule OR GPT-4	1.00	0.81	0.89	0.81
Perianal—no	Rules	0.97	1.00	0.98	1.00
	GPT-4	0.98	0.98	0.98	0.98
	Rules AND GPT-4	1.00	1.00	1.00	1.00
	Rule OR GPT-4	0.95	1.00	0.98	1.00

Stricturing and penetrating complications were combined into one “B2/B3” category. Rules AND GPT-4: Only instances with the same results from rules and GPT-4 are considered; Rules OR GPT-4: disease complication was considered if labeled by either rules or GPT-4.

Using radiology reports, the model performance values drop to 0.64– 1.00, with less sensitive identification, particularly of B3 and B2 (Table A.7). The underlying reason may be the incorrect identification of B2 or B3 as perianal disease. The precision values and F1 scores indicate over-classification, resulting in false positive disease complication labels.

Combining both approaches, if at least labeled by one of the two approaches (Rules OR GPT-4), we improve recall for detection of disease complications (1.00). However, we lose precision (0.81 for perianal disease, 0.78 for structuring or penetrating disease) (Table 1). Only considering instances with the same labels based on the rules and GPT-4 yields the highest performance metrics with balanced recall and precision, with F1 scores between 0.97 and 1.00. The overlap between labels derived from the GPT-4 model and the rules for perianal disease is at 95%, for Not B2/B3 and B2/B3 at 90%.

### Joint evaluation of rules and GPT-4 enables chart-review prioritization

For 134 patients in the MSCCR study, we extracted disease phenotype at study enrollment through manual chart review, considering all clinical information up until this point. Compared to the note-level analysis, using the rule-based approach, we achieve a recall value of 0.71 and a precision of 0.48 for detecting any complication (B2 or B3). For the detection of perianal disease, the recall is 0.85, and the precision is 0.56, both of which decrease compared to the note-level analysis (Table 2).

**Table 2:** Performance of rule-based phenotyping algorithms and in-context learning using a GPT4 model to extract disease behavior on the patient level

Phenotype	Model	Recall	Precision	F1 score	Specificity
Not B2/B3	Rules	0.65	0.83	0.73	0.48
	GPT-4	0.86	0.84	0.85	0.68
	Rules AND GPT-4	0.87	0.83	0.85	0.72
	Rule OR GPT-4	0.64	0.83	0.72	0.48
B2/B3	Rules	0.71	0.48	0.58	0.83
	GPT-4	0.64	0.68	0.66	0.84
	Rules AND GPT-4	0.66	0.72	0.69	0.83
	Rule OR GPT-4	0.71	0.48	0.57	0.83
Perianal—yes	Rules	0.85	0.56	0.68	0.56
	GPT-4	0.78	0.58	0.67	0.58
	Rules AND GPT-4	0.84	0.68	0.75	0.68
	Rule OR GPT-4	0.85	0.50	0.63	0.50
Perianal—no	Rules	0.83	0.96	0.89	0.96
	GPT-4	0.86	0.94	0.90	0.94
	Rules AND GPT-4	0.89	0.95	0.92	0.95
	Rule OR GPT-4	0.79	0.95	0.86	0.95

In total, 134 patients of the MSCCR cohort had available information on the behavioral disease phenotype through manual chart review. Stricturing and penetrating complications were combined into one “B2/B3” category. Rules AND GPT-4: Only instances with the same results from rules and GPT-4 are considered; Rules OR GPT-4: disease complication was considered if labeled by either rules or GPT-4.

Also, for the GPT-4-based approach, the performance drops compared to the note-level analysis. Similar to the note-level results, aggregating stricturing (B2) and penetrating (B3) disease into one disease complication category (B2/B3) improves results (Table A.8).

As in the note-level evaluation, we apply ensemble methods based on the combined output from the two approaches. This includes either focusing only on patients with the same labels from both methods (Rules AND GPT-4, representing 77% overlap for B2/B3 and 89% for perianal disease), or considering a disease complication if detected by either method (Rules OR GPT-4). Only considering overlapping labels yields a better balance between recall and precision, with F1 scores of 0.69 (B2/B3) and 0.75 (perianal disease).

The GPT-4 model alone performs well at identifying the non-existence of disease complications (no B2/B3 and no perianal disease), with an F1 score of 0.85 and 0.90, respectively. This reflects a reduction in false positives compared to the rule-based approach. For perianal disease, the F1 score is further improved by 0.02 using the overlap ensemble approach.

We evaluate the count of note-level labels per patient sub-group to better understand whether a count-based cut-off may help to further stratify patients for chart review. Patients labeled as B2/B3 or perianal disease have a significantly higher number of notes in their records classified with the respective condition compared to patients without any labeled complication (Figs. A.6 and A.7). However, for a considerable number of patients (rules: 15, GPT-4: 12) with known disease complications, this information is not extracted from or noted in any of the clinical notes. Therefore, stratification based on the note count of extracted complications does not remain a suitable option.

Of note, for the annotation process of the patient-level ground-truth labels, as the primary clinical information system is used, the underlying data differs from the information available for automated phenotyping.

### GPT-4 outperforms the rule-based approach in age at diagnosis extraction

We evaluate the performance of the age at diagnosis extraction through the rules and GPT-4 model on note- and patient-level (Table 3). Using the rule-based approach, we observe balanced performance metrics on the note-level, exemplified by a recall of 0.78 and a precision of 0.87. The GPT-4 model outperforms the rule-based model with a recall of 1.00 and a precision value of 0.87. The correlation between the extracted ages at diagnosis is very high with 0.98 for GPT-4 (Fig. A.8). While also showing a higher correlation compared to the rule-based approach (*R* = 0.88), the GPT-4 model is also able to label all notes that include information on the age at diagnosis (64 out of 80). In contrast, the rules only recognize 79% of the notes. Aggregating the results from GPT-4 and the rule-based approach by calculating the mean or only including instances where the extracted age at diagnosis is the same between rules and GPT-4 does not considerably improve overall results.

**Table 3:** Performance of rule-based phenotyping algorithms and in-context learning using a GPT4 model to extract age at diagnosis on note- and patient-level

Phenotype	Evaluation	Model	Recall	Precision	F1 score	Specificity
Age at diagnosis	Note-level	Rule-based	0.78	0.87	0.82	0.68
		GPT-4	1.00	0.87	0.93	0.53
	Patient-level	Rule-based	0.73	0.70	0.71	NA
		GPT-4	1.00	0.59	0.74	NA

Eighty clinical notes were newly annotated for evaluation of this task. Patient-level performance evaluation was conducted using 458 MSCCR patients with available clinical narrative texts and existing age at diagnosis labels of the cohort. NA values are based on the fact that all patients have an annotated age at diagnosis, resulting in 0 true negatives.

On the patient level, a similar balance of the performance measures is achieved using the rule-based approach, with lower overall performance values compared to the note level, with F1 scores of 0.71 and 0.82, respectively. Conversely, the metrics from GPT-4 are not as balanced, with a recall of 1.00 and a precision of 0.59, but overall a higher F1 score (0.74) compared to the rule-based approach. The correlation of GPT-4 and the rule-based age at diagnosis with the manually labeled values is high (GPT-4: *R* = 0.84; Rules: *R* = 0.81), but reduced compared to the note-level (Fig. A.9). Only considering patients with the same labels yields an *R* value of 0.86, but compared to the GPT-4 based approach, a reduction of extracted labels by 34%.

The underlying data of the 458 MSCCR patients for evaluation at the patient level are not specifically scanned for the availability of the information in scope within the written clinical text. For instance, colonoscopy reports and pathology reports are not accessible for this study, but are, according to the coded procedures, often conducted similarly to radiology procedures (Fig. A.10). These missing reports may contain relevant information for the described disease characteristics. Therefore, the reduced performance of the model at the patient level may be explained by the limited data availability.

## Discussion

In the current study, we demonstrate the feasibility of automatically extracting clinical information on CD disease behavior and age at diagnosis from EHR using a rule-based and LLM-based approach. In particular, the dynamic nature of the disease behavior variable can be captured for longitudinal analyses using our approach. In addition to the labeling support it offers for research purposes, the integration into clinical workflows and existing hospital systems, such as EPIC, could facilitate clinical decision support and real-time coding. For instance, usage of such a tool may help identify patients with complicated disease behavior who are not on advanced therapies, and facilitate their referral for IBD expert consultation. As a first step toward clinical implementation, we provide highlighted texts showing the matched spans to facilitate and accelerate labeling tasks. To our knowledge, we are the first to describe NLP-based phenotyping for the stated tasks. GPT-4 performs as well or better than the rule-based approaches. Our results suggest that, compared to rules, large general-purpose language models may provide a more efficient and better-performing method for automated disease phenotyping in IBD.

The IAA of manual chart review of the described phenotypes differs between previous studies, with Cohen’s kappa values between 0.54 and 0.79 for disease behavior and between 0.67 and 0.98 for age at diagnosis^22–24^. These findings underscore the complexity of both tasks, particularly for the extraction of disease behavior. To evaluate our algorithms, we create two datasets annotated at the sentence level. For disease behavior, we include two annotators. with an IAA on average between 0.85 and 0.90, surpassing the kappa statistics from previous studies. The lower end of the previously reported values in the literature illustrates the substantial subjectivity in disease behavior annotation, which may impact model training and evaluation. Of note, the sentence- or note-level annotation agreements from our analysis are difficult to compare with patient-level annotations from previous studies. In contrast to note-level annotations, patient-level phenotyping requires integrating data across multiple longitudinal data sources, including imaging, endoscopy procedures, and surgeries. This results in significantly larger datasets, where critical information about disease phenotypes may be unevenly distributed. This increased volume raises the risk of misclassifications, for instance, due to overlooked but relevant information—an issue that can affect both manual review and computable phenotyping approaches.

Shrestha et al. describe the identifications of disease phenotypes using International Classification of Diseases (ICD)-codes of the Swedish National Patient Register^21^. For our use case, working with ICD-codes is not a suitable option: Given the fragmented nature of the US healthcare system^33^, coded information in EHR data is not sufficiently reliable to extract the complex clinical information^7^. Nevertheless, the published study by Shrestha et al. provides a baseline for computable phenotyping with which we can compare our patient-level results. Their reported recall values lie between 0.62 for B2/B3, 0.75 for B1, and 0.81 for perianal disease, and on average, 0.94 for the different phenotype groups of age at diagnosis. Our results are overall comparable when only considering patients with identical labels using the rules and GPT-4, even exceeding the results reported based on the ICD codes from the Swedish Patient register. Furthermore, our reported performances are similar to those described for other tasks in the literature, such as the extraction of extraintestinal manifestations^20^.

The presented rule-based models offer systematic and transparent reasoning and are thus potentially the preferred support for labeling tasks in a clinical setting. For disease behavior, the general-purpose LLM, GPT-4, achieves similar results as the rule-based approach. The model’s capacity to digest and consider the context of one clinical note as a whole is of interest for context-dependent phenotyping tasks. Particularly for age at diagnosis, the recall of the GPT-4 model with 1.00 on note- and patient-level is considerably higher than for the rule-based approach with 0.78 and 0.73, respectively. While on the patient-level, this increased value is accompanied by a reduction of precision by 0.11, the extraction of time or basic demographic information may be an easier task than the extraction of medical domain-specific information for a general LLM such as GPT-4^34^. In addition, an important consideration is the time effort required to develop the different approaches: the estimated time effort to develop the rules for disease behavior, including the development of the task-specific UMLS subset for matching, is about two months of full-time work and for the rules for age at diagnosis extraction, an additional 2 weeks. The implementation and development of the prompt for GPT-4 takes about a day. Our approach to incorporating the annotation guidelines into the prompt, in addition to randomly selected examples from the development set, provides a quick and successful solution for prompt engineering. An additional consideration is infrastructure cost. The rule-based approach requires minimal computational resources once developed and can be deployed on local infrastructure. In contrast, deploying large language models such as GPT-4, especially at scale, may lead to substantial computational and financial costs, particularly if relying on commercial application programming interfaces (APIs). Another critical factor is data privacy. While rule-based models can be run locally within secure health system environments, GPT-4’s current usage often depends on cloud-based APIs. Even though applied here in a HIPAA-compliant manner, this introduces potential privacy concerns, as clinical text must leave the local system unless the model is available for on-premise deployment.

For a more in-depth understanding of false classifications on the note-level, we analyze all false classifications from the rule-based approach and the LLM approach for the behavioral phenotyping (Tables A.9 and A.10). Additionally, on the patient level, we analyze five falsely positive and falsely negative classified patients for B2/B3 and perianal disease from the rule-based approach. False positive instances for B2/B3 mainly come from describing similar complications in other disease contexts (e.g., carotid stenosis), complex sentence structures leading to errors in negation detection, and confusion with perianal disease labels. Instances misclassified as perianal disease are suspected to be partly wrong-labeled. In one instance, the negation detection is not sophisticated enough to catch the negation in the given sentence structure. There is no clear description of the phenotype in the clinical texts for patients with false negative labels of B2/B3 and perianal disease. Challenges for the age at diagnosis extraction are mainly caused by the varied representations of dates in the data, coupled with the task of unambiguously linking a date occurrence to the diagnosis of CD.

On a patient level, an error analysis through manual chart review underscores the complexity of the disease behavior phenotype extraction from unstructured clinical notes. Contextual distinctions (e.g., inflammatory vs. fibrotic stenosis) or ambiguous language (e.g., inactive vs. active complications), which can be challenging even for human annotators, are often likely the source of error for the algorithmic approaches. Of note, a few examples labeled as not perianal disease and not B2/B3 by the algorithms, but labeled with disease complications by the human annotator, turn out to be true negatives at the time point of MSCCR enrollment. The error likely stems from the mention of disease complications after baseline. These findings underscore both the promise and limitations of current NLP approaches: while not infallible, they can flag potentially overlooked information and serve as valuable aids to manual chart review, particularly for phenotypes like B2/B3 or perianal disease, where interpretation is context-heavy and temporally dynamic.

While our study shows promising results, we acknowledge certain limitations. Foremost, due to the fragmentation of clinical data into multiple IT systems, we do not have access to endoscopy or pathology reports for our study. Adding these data types may improve future studies. Furthermore, our phenotyping pipeline is based only on information captured in clinical text and does not incorporate structured EHR data. With this underlying difference of utilized data for computable phenotyping compared to manual chart review, and without checking whether the information we are looking for is, in fact, captured in our underlying data, the patient-level evaluation has to be regarded with caution. Prioritization of patients for chart review may be one future application in order to accelerate the labeling process and improve patient quality. Patients labeled as non-penetrating, non-stricturing and non-perianal disease by the two approaches may be reviewed with less time spent, as the automated phenotyping performs very well in these cases. Overall, phenotyping results at the patient level may overestimate the number of complications when aggregating across multiple notes. This should be considered when interpreting cohort-level disease progression over time. Moreover, we are not able to assess temporal changes in phenotypes at the individual level, as this would require a dataset with temporally resolved annotations. Our current aim is to determine how accurately the models can assign Montreal classification based on the totality of clinical notes, consistent with the classification’s use of maximal disease behavior. Future studies should assess the ability of such tools to assign the timing of phenotype evolution and evaluate their performance in longitudinal phenotyping.

As a second limitation, our models are only evaluated on internal clinical texts from Mount Sinai Health System (MSHS). While the dataset includes data from six hospitals and reflects variation in documentation practices, these remain institution-specific. Broader validation on external datasets or independent expert annotations would strengthen confidence in the extracted phenotypes and is needed to assess the generalizability of our approaches. In a clinical context, it is imperative that future research addresses the potential biases introduced by the models and their effect on patient care^35^.

Furthermore, we did not conduct a systematic prompt sensitivity analysis, but iteratively refined the prompt on the development set to maximize clarity and alignment with the annotation rules. While recent literature suggests that prompt engineering can affect absolute performance levels, it typically does not change the relative ballpark comparison across fundamentally different approaches (e.g., rule-based vs. LLM-based)^36–38^.

Lastly, our study does not compare GPT-4 with other domain-specific LLM or named entity recognition (NER) models beyond our rule-based baseline. While both the rule-based methods and off-the-shelf GPT-4 perform strongly without fine-tuning, including additional models would enhance the completeness of the evaluation. However, recent studies demonstrate that static, one-off cross-model benchmarks on rapidly evolving LLM can become non-repeatable and quickly outdated, particularly as vendors update models and variants over time^39^. For the same reasons, we also did not include open-source LLM (e.g., LLaMA-based models). Recent research shows that such models can perform comparably to commercial LLM^40–42^.

While the phenotypes in our study are often explicitly stated in the text, future work may also explore supervised machine learning models to improve robustness against different clinical documentation styles and allow for improved generalizability^43^. Such models may be particularly valuable for capturing indirect mentions of disease behavior and directly learn features from both structured and unstructured data.

## Conclusion

In conclusion, GPT-4 performs at least comparable and, in some cases, better than the rule-based approach for CD disease phenotyping, with little effort involved. Therefore, we anticipate that LLMs will be increasingly deployed for phenotyping tasks and that our work can contribute to studies based on large patient cohorts. An immediate application may, for instance, lie in the prioritization of patients for manual chart review. External validation and benchmarking against other NER models and domain-adapted LLMs such as MEDITRON or BioMistral would help establish broader applicability and robustness of the phenotyping approach^44,45^.

## Supplementary information

Supplementary Info

## Data Availability

The data used for this study contains protected health information (PHI) and cannot be shared publicly due to patient privacy reasons.

## Code availability

The underlying code for this study is available on GitLab and can be accessed via this link: https://gitlab.com/dacs-hpi/cd-nlp-phenotyping^46^.
