# Supplementary material for: Automating clinical phenotyping using natural language processing
Source: Commun Med (Lond). 2026 Jan 14;6:77. doi: 10.1038/s43856-025-01337-0 (PMC12873203; doi:10.1038/s43856-025-01337-0)
Supplement: Supplementary file 1 — Supplementary Info [file 43856_2025_1337_MOESM1_ESM.pdf]

Appendix A. Supplementary Material

Appendix A.1. Supplementary Tables

Table A1: Montreal Classification for Crohn's Disease patients according to Silverberg *et al.*[10]. † "p" is added to B1–B3 when concomitant perianal disease is present. *L4 is a modifier that can be added to L1–L3 when concomitant upper gastrointestinal disease is present.

**Category**	**Classification**	**Definition**
Age at Diagnosis	A1	Below 16 years
	A2	Between 17 and 40 years
	A3	Above 40 years
Disease Behavior	B1	Non-stricturing and non-penetrating
	B2	Stricturing
	B3	Penetrating
	p†	Perianal disease modifier
Disease Location	L1	Ileal
	L2	Colonic
	L3	Ileocolonic
	L4*	Isolated upper disease

Table A2: Overview of the annotation process of the test dataset for the behavioral disease phenotype using clinical notes and radiology reports.

		Clinical Notes	Radiology Reports	Total
**Total number of notes**		150	50	200
**Total number of sentences**		14,236	1,154	15,390
**Mean sentences per note (SD)**		95 (±87)	23 (±11)	-
**Not B2/B3**	**Notes**	112	32	144
	**Sentences**	14,094	1,113	15,207
**B2**	**Notes**	13	7	20
	**Sentences**	62	24	86
**B3**	**Notes**	25	11	36
	**Sentences**	80	17	97
**Perianal disease**	**Notes**	25	7	32
	**Sentences**	113	24	137

Table A3: Cohen's kappa agreement scores calculated as inter-annotator agreements (IAA) between the two annotators on sentence- and note-level, as well as between the consensus labels from the two annotators, and the labels derived using GPT-4 or the rules on note-level, respectively.

**Comparison**	**Not B2/B3**	**B2/B3**	**Perianal**	**Average**
Sentence-level IAA	0.83	0.84	0.87	0.85
Note-level IAA	0.88	0.88	0.95	0.90
GPT-4/consensus IAA	0.80	0.80	0.89	0.83
Rules/consensus IAA	0.84	0.84	0.90	0.86

Table A4: Overview of the annotation process of the test dataset for the age at diagnosis using clinical notes and radiology reports.

		Clinical Notes	Radiology Reports	Total
**Total number of notes**		79	1	80
**Total number of sentences**		12,261	32	12,293
**Mean sentences per note (SD)**		155 (±116)	32 (NA)	-
**Age at diagnosis**	**Notes**	18	0	18
	**Sentences**	21	0	21
**Year of diagnosis**	**Notes**	47	1	48
	**Sentences**	76	1	77
**Disease duration**	**Notes**	8	0	8
	**Sentences**	10	0	10

Table A5: Performance of the rule-based phenotyping algorithm and GPT-4-based results of disease behavior phenotyping on note level using the newly annotated test dataset comprising in total 200 clinical notes and 50 radiology reports.

Model	Phenotype	Recall	Precision	F1 score	Specificity
Rule-based	Not B2/B3	0.94	1.00	0.97	1.00
Rule-based	B2	0.92	0.75	0.83	0.97
Rule-based	B3	1.00	0.86	0.93	0.97
GPT-4	Not B2/B3	0.95	0.98	0.96	0.95
GPT-4	B2	0.92	0.57	0.71	0.93
GPT-4	B3	0.80	0.95	0.87	0.99

Table A6: Statistical differences between the Cohen's kappa agreement scores from the note-level inter-annotator agreement (IAA) of the two annotators, as well as between the consensus labels from the two annotators, and the labels derived using GPT-4 or the rules on note-level, respectively. Differences are reported in form of the Z-statistic and the corresponding p-values and q-values (FDR-adjusted p-values).

	GPT-4 vs note-level IAA			Rules vs note-level IAA		
**Phenotype**	**Z-score**	**p-value**	**q-value**	**Z-score**	**p-value**	**q-value**
Not B2/B3	-0.74	0.46	0.70	-0.39	0.70	0.70
B2/B3	-0.74	0.46	0.70	-0.39	0.70	0.70
Perianal	-0.78	0.43	0.70	-0.67	0.50	0.70

Table A7: Performance of the rule-based phenotyping algorithm and GPT-4-based results of disease behavior phenotyping on note level using the newly annotated test dataset comprising 50 radiology reports.

Model	Phenotype	Recall	Precision	F1 score	Specificity
**Rule-based Approach**	**Not B2/B3**	0.91	0.94	0.92	0.89
	**B2**	0.71	0.50	0.59	0.88
	**B3**	0.64	0.78	0.70	0.95
	**p - Yes**	1.00	0.80	0.89	0.80
	**p - No**	0.95	1.00	0.98	1.00
**GPT-4-based Approach**	**Not B2/B3**	0.81	1.00	0.90	1.00
	**B2**	1.00	0.47	0.64	0.81
	**B3**	0.64	0.78	0.70	0.95
	**p - Yes**	1.00	1.00	1.00	1.00
	**p - No**	1.00	1.00	1.00	1.00

Table A8: Performance of the rule-based phenotyping algorithm and GPT-4-based results of disease behavior phenotyping on patient level. 134 patients of the MSCCR cohort had available information on the behavioral disease phenotype through manual chart review.

Model	Phenotype	Recall	Precision	F1 score	Specificity
Rule-based	Not B2/B3	0.65	0.83	0.73	0.71
Rule-based	B2	0.54	0.41	0.46	0.83
Rule-based	B3	0.61	0.37	0.46	0.84
GPT-4	Not B2/B3	0.86	0.84	0.85	0.64
GPT-4	B2	0.58	0.50	0.54	0.87
GPT-4	B3	0.50	0.75	0.60	0.97

Table A9: Missclassifications on note-level for the rule-based phenotyping approach.

Clinical Text	Label	NLP-Result	Explanation
"obstructive sleep apnea, [...], hypertension, inflammatory bowel"	Not B2/B3	B2/B3	"obstructive [...] bowel" matched b2_string matcher
"was taken to the operating room and had fistulotomy of a left, lateral fistula and placement of a seton in an additional posterior fistula and presents now for removal of the seton and closure of the fistula."	Not B2/B3	B2/B3	From note context: reference to perianal fistula
"crohns with recent dx of rectal abscess [...] large swollen rectal area a.p cocnern for recurrence of abscess, retention is likely due to pressure, but fistulas and medication effect cannot be ruled out."	Not B2/B3	B2/B3	"fistula" just suspected
"anal fissure still was not healed [...] saw [...] for a lump in their anal area. was told that had an abscess [...] when they saw fistula they did a fistulectomy"	Not B2/B3	B2/B3	From note context: reference to perianal fistula
"with evidence for multiple areas of beading/irregularity, and strictures involving the biliary bifurcation, compatible with PSC."	Not B2/B3	B2/B3	b2_matcher and b2_string_matcher match "strictures" to B2
"denies peptic ulcer disease, jaundice, liver diseases currently, gallstones, melena, anal fissures, and all fistulae."	no p	p	Perianal disease match since "denies" is located to far from the match occurrence
"colonoscopy revealed mild crohns' colitis with apthous ulcers from the rectum to the proximal colon; the anastomosis was narrowed and could not be traversed."	no p	p	"anastomosis" matched CUI of "Anastomosis of rectum"
"no rash, clubbing, cyanosis, or edema imp/severe fistulizing sb crohn's, s/p 2nd icr [...] s/p takedown [...]."	no p	p	perianal disease match of "fistulizing sb crohn's"
"fistulizing crohn's disease [...] with recent mre showing enteroenteric and enterocolonic fistulas now s/p icr with re-anastamosis"	no p	p	"enterocolonic fistulas" matched CUI of "Anal Fistula"

Table A10: Missclassifications on note-level for the GPT phenotyping approach. Upon manual review, the presented clinical text snippets appear to be the segments of the clinical notes most likely contributing to the misclassification.

Clinical Text	Label	GPT-Result	Comment
"large swollen rectal area A.P cocnern for recurrence of abscess, [...] but fistulas and medication effect cannot be ruled out."; *"multiple I and D for left and right ischiorectal abscess and horseshoe abscess."*	Not B2/B3	B2/B3	
"Rectal Exam: Normal rectal exam. Stricture."; *"Findings: Rectal stricture and nodularity. Sigmoid pseudopolyps. Edema at anastamosis."*	Not B2/B3	B2/B3	
*Gastrointestinal: Crohnś disease, S/P ileocolic resection (*year*)*; *Abdomen MRI: no hepatic malignancy, [...], mild left intrahepatic bile duct stricturing*	Not B2/B3	B2/B3	
???	Not B2/B3	B2/B3	
*past medical history of Crohn's s/p ileocecectomy on *date* pod 1.*	Not B2/B3	B2/B3	
history of Crohn's disease and presented elsewhere with an abscess and subsequently a fistula. [...] Seton placed elsewhere. [...] known to have Crohn's colitis with stricturing in 2 places	B2/B3	Not B2/B3	
NO EVIDENCE OF BOWEL OBSTRUCTION. NO DEFINED COLLECTION/ABSCESS.; *Impression: Several loops of distal ileum demonstrating circumferential wall thickening.*; *MRE showed distal ileum wall thickening with small bowel mesentery edema*	Not B2/B3	B2/B3	
severe chronic active colitis with acute cryptitis and crypt abscesses.	B2/B3	Not B2/B3	inconclusive description - abscess could refer to known perianal disease.
Mild distal rectal stricture.	p	no p	
Ankylosing spondylitis /crohnś on clizia C/o increase rectal bleeding	no p	p	
Medications: MAC Anesthesia Rectal Exam: Normal rectal exam. Stricture.; *Findings: Rectal stricture and nodularity.*	p	no p	
Lidocaine-Hydrocortisone Ac (ANAMANTLE HC) 3-0.5 % kit Use rectally twice daily	no p	p	

Appendix A.2. Supplementary Figures

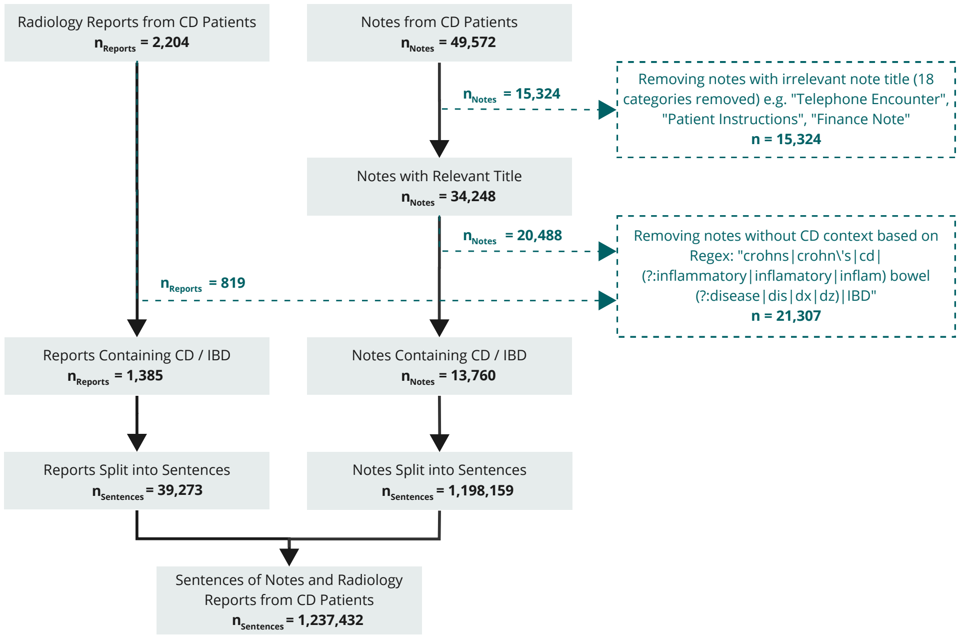

Figure A1: Data sources and preprocessing steps. After extracting all available clinical notes from CD patients in MSCCR up until two weeks after the date of initial endoscopy and biopsy for sample collection for the study, all notes with irrelevant tiles were removed. Subsequently, from the available clinical notes and radiology reports, only disease-relevant texts were further processed by splitting them into sentences.

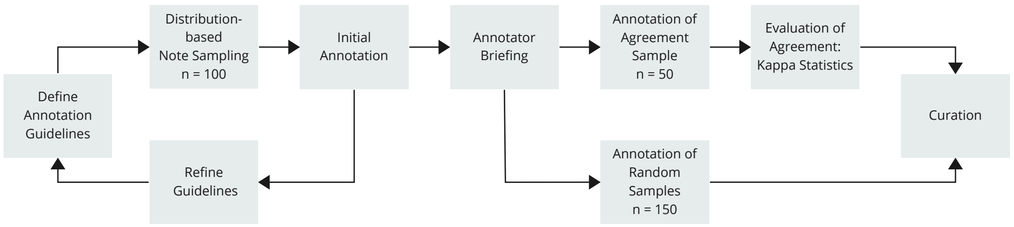

Figure A2: Labeling process. The process from building annotation guidelines to a final annotated and curated dataset for CD disease complications containing 150 clinical notes and 50 radiology reports.

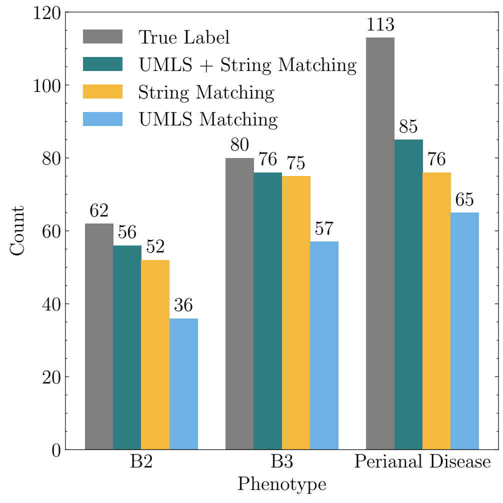

(A) Clinical Notes

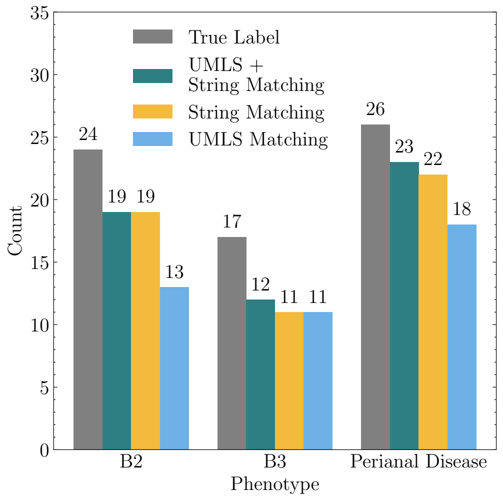

(B) Radiology Reports

Figure A3: Correctly identified phenotypes by patterns. The count of correctly identified phenotypes by different usage of patterns versus the true count per phenotype on sentence level in the (A) annotated clinical notes and (B) radiology reports. UMLS matching refers to patterns using the matched UMLS codes, while string matching refers to patterns manually created to match specific phenotypes.

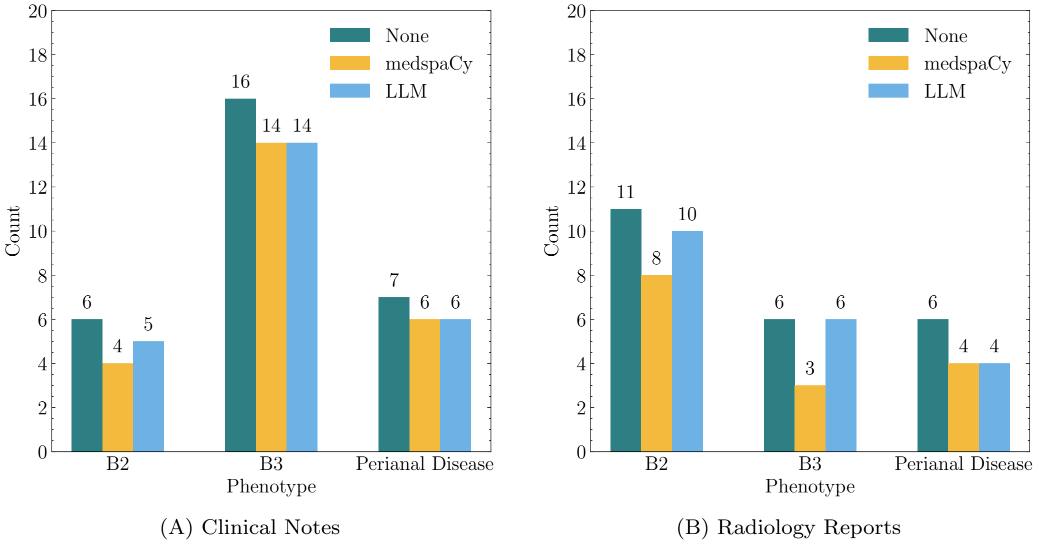

Figure A4: Incorrect identified phenotypes by negation detection methods on sentence-level. Differences in negation detection methods when analyzing the count of incorrectly identified phenotypes in the (A) annotated clinical notes and (B) radiology reports. "None" means, that no additional negation detection besides the manually defined rules for uncertainty and exclusion is used. "medspaCy" refers to negation detection based on the medspaCy ConText component, and "LLM" refers to negation detection via the clinical-assertion-negation-bert classifier.

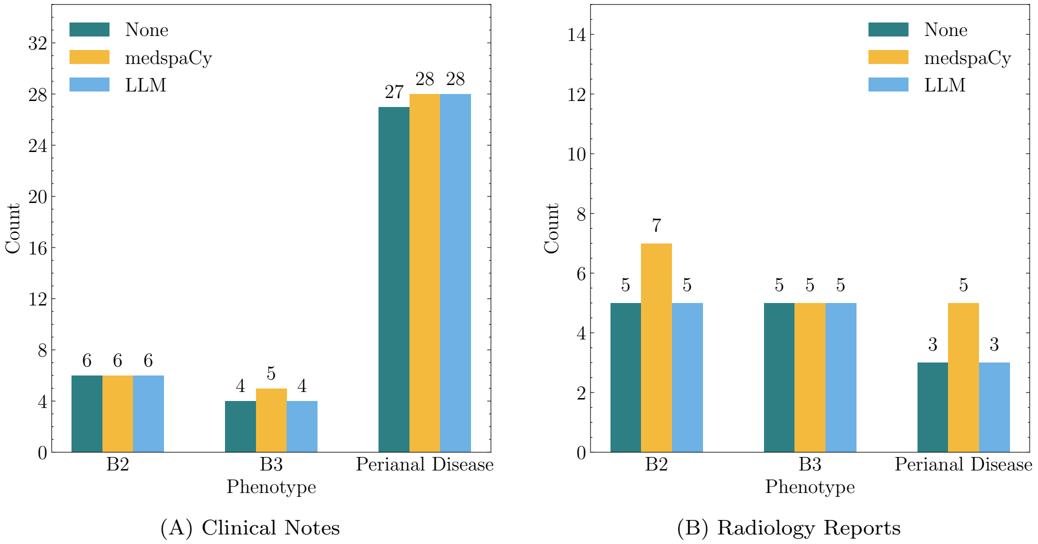

Figure A5: False negative phenotypes by negation detection methods. Differences in negation detection methods when analyzing the count of false negative phenotypes in the (A) annotated clinical notes and (B) radiology reports. "None" means, that no additional negation detection besides the manually defined rules for uncertainty and exclusion is used. "medspaCy" refers to negation detection based on the medspaCy ConText component, and "LLM" refers to negation detection via the clinical-assertion-negation-bert classifier.

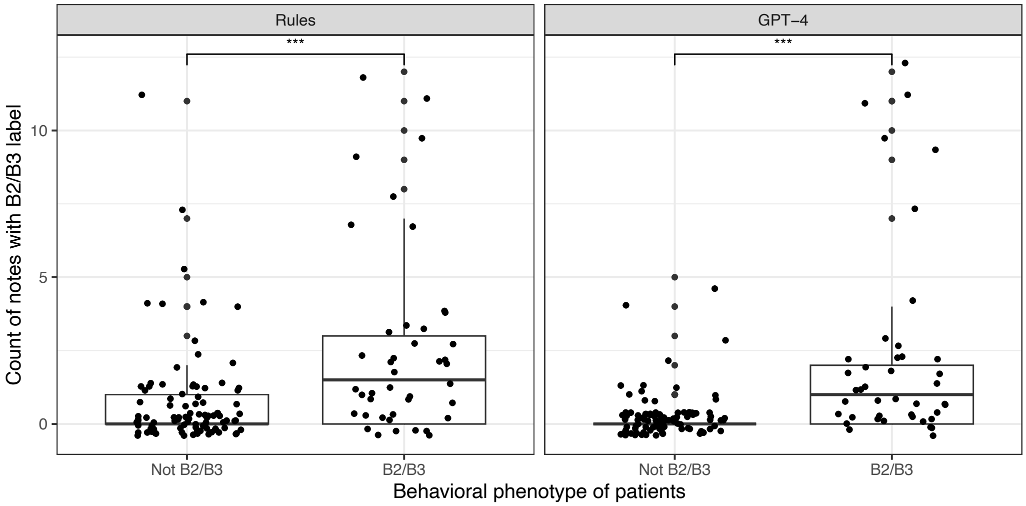

Figure A6: Counts of as penetrating disease classified notes per patient and phenotype group. 24 of the 134 labeled patients had stricturing disease (B2) and 18 penetrating disease (B3) at time point of study enrollment. Difference in note count between the groups was calculated using the Wilcoxon rank-sum test. NS.: not significant; "*": p-value < 0.05; "**": p-value < 0.01; "***": p-value < 0.001.

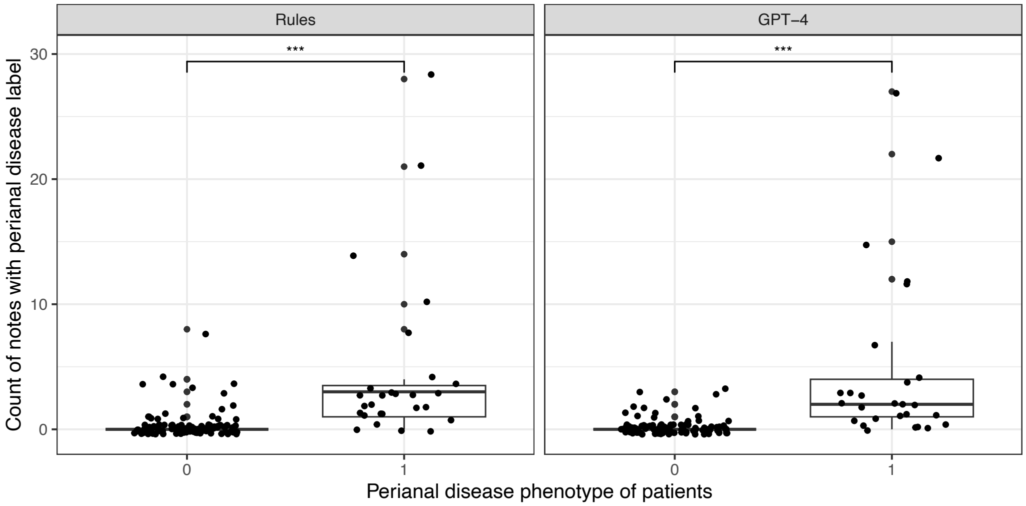

Figure A7: Counts of as perianal disease classified notes per patient and phenotype group. 27 of the 134 labeled patients had perianal disease at time point of study enrollment. Difference in note count between the groups was calculated using the Wilcoxon rank-sum test. NS.: not significant; "*": p-value < 0.05; "**": p-value < 0.01; "***": p-value < 0.001.

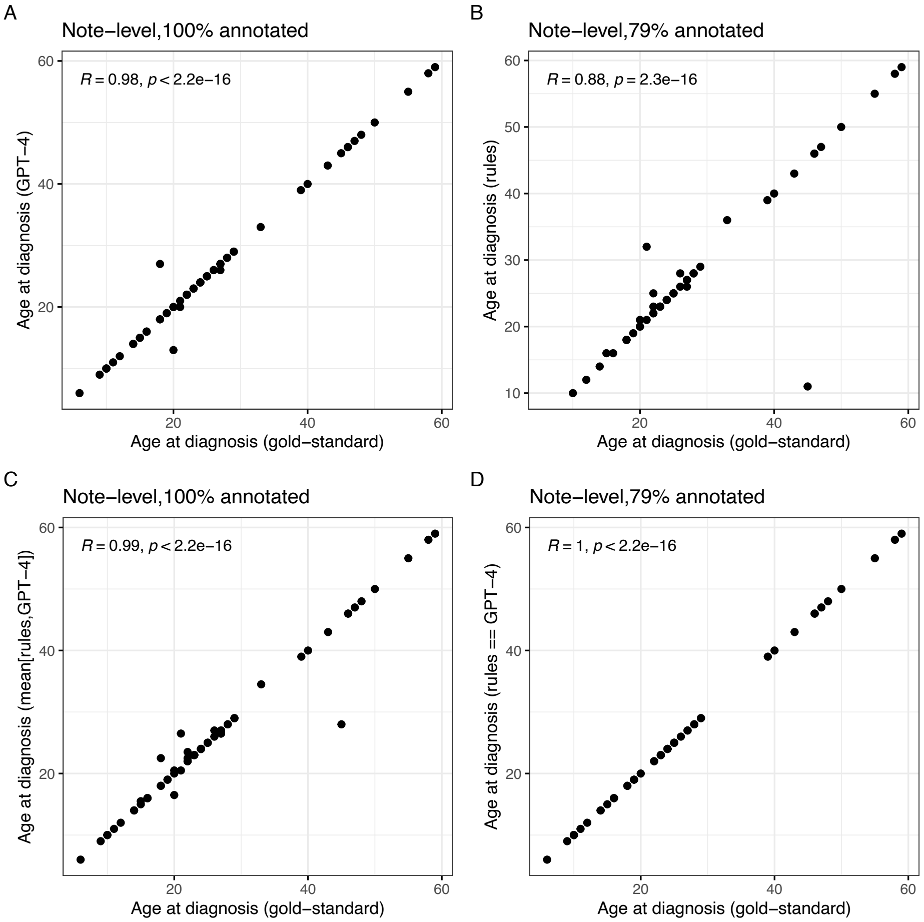

Figure A8: Pearson correlation of ground-truth age at diagnosis derived manual annotation and automatically extracted age at diagnosis values on note-level. Automatic extraction of age at diagnosis included (A) a GPT-4-based approach, (B) a rule-based approach, (C) a combination of the two, choosing the mean age at diagnosis value, and (D) the subset of notes where the extracted age at diagnosis value through rules and GPT-4 was identical. Fraction of notes that were annotated by each approach given in the header title.

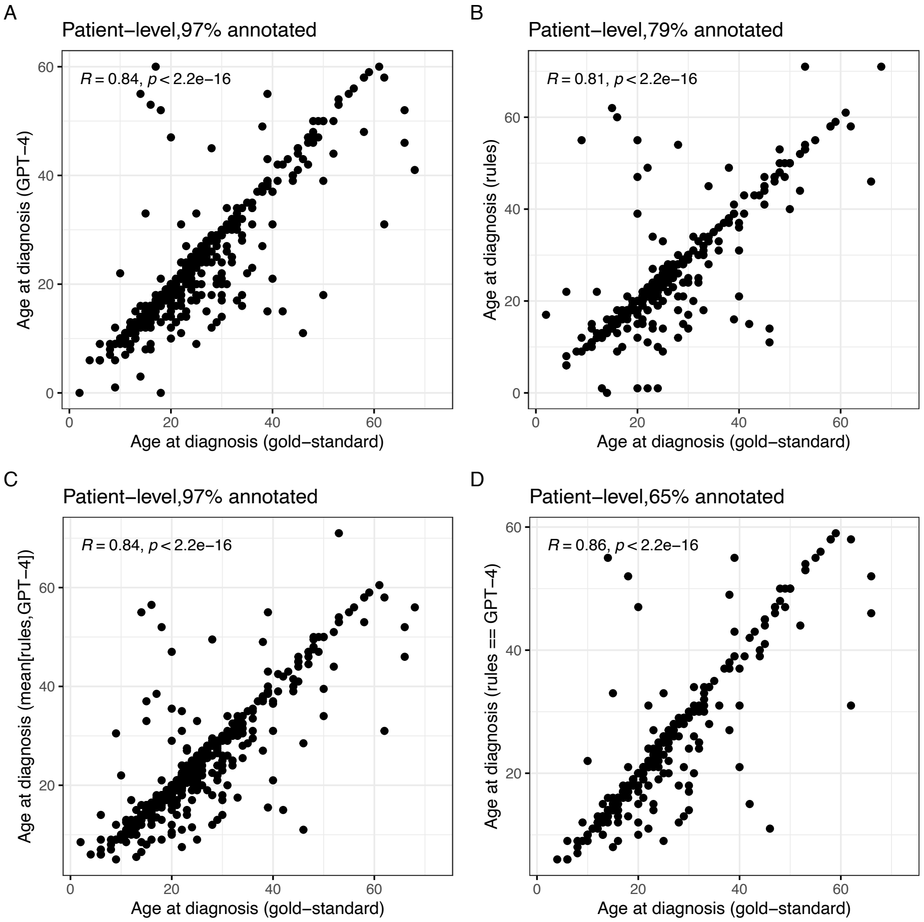

Figure A9: Pearson correlation of ground-truth age at diagnosis derived manual annotation and automatically extracted age at diagnosis values on patient-level. Automatic extraction of age at diagnosis included (A) a GPT-4-based approach, (B) a rule-based approach, (C) a combination of the two, choosing the mean age at diagnosis value, and (D) the subset of notes where the extracted age at diagnosis value through rules and GPT-4 was identical. Fraction of patients that were annotated by each approach given in the header title.

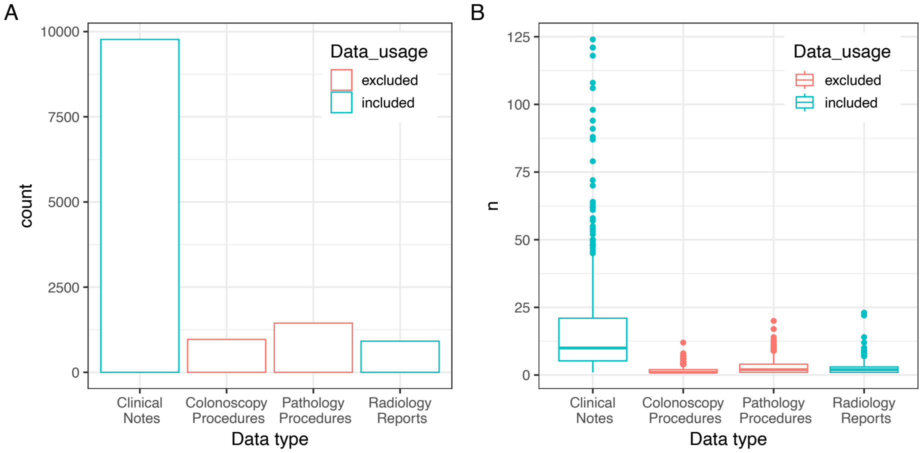

Figure A10: Overview of available and missing clinical data across different sources in the MSCCR dataset. Endoscopy and pathology reports were not directly accessible for this analysis; instead, counts were estimated based on coded procedures in the structured electronic health records. (A) Histograms show the total number of unique clinical texts or procedures across the entire dataset. (B) Boxplots display the distribution of unique clinical texts or procedures per patient.

Appendix A.3. Supplementary Methods

Appendix A.3.1. Regular Expressions for Age at Diagnosis Notes Filtering

Included clinical texts needed to match at least one of the following patterns:
`(D|d)iagnosed|DIAGNOSED`,
`((C|c)rohn|CROHN|cd|CD)[^a-zA-Z0-9]*(since|SINCE)`,
`(D|d)isease[^a-zA-Z0-9]*(O|o)nset|DISEASE[^a-zA-Z0-9]*ONSET`,
`(A|a)ge[^a-zA-Z0-9]*(A|a)t[^a-zA-Z0-9]*(D|d)iagnosis`,
`AGE[^a-zA-Z0-9]*AT[^a-zA-Z0-9]*DIAGNOSIS`

Appendix A.3.2. Rule-based Disease Behavior Phenotyping

A custom spaCy component, BehavioralPhenoCategorizer, was constructed for phenotype extraction. After preprocessing, abbreviation detection, and UMLS linking using a curated subset of UMLS Metathesaurus codes, patterns were established to detect specific CD behavioral phenotypes. The development process utilized spaCy's Matcher class to design patterns that describe token sequences for accurate disease phenotyping of CD. Multiple patterns were crafted: for specific phenotype complications, direct string-level matches, UMLS linkages, and two additional patterns addressing medical conjectures (uncertainty matcher) and explicit exclusions (exclusion matcher). These patterns were refined to differentiate B2 and B3 complications from perianal disease through UMLS linking and token-level regular expression-like patterns.

In clinical texts, the presence and absence of medical conditions are often described, making effective negation detection crucial. For behavioral phenotyping in CD, two strategies were adopted: one leveraging medspaCy, a rule-based approach that identifies negation patterns and uses dependency parsing to determine negated entities, and the other utilizing a Transformer-based Clinical Assertion and Negation Classification BERT model [29]. For the latter, we deployed the pre-trained *bvanaken/clinical-assertion-negation-bert* model from the Hugging Face Hub[2], considering spans as negated if they surpass a probability score threshold of 0.5.

The BehavioralPhenoCategorizer processed each document in stages (Figure 2): initial categorization using UMLS matching, pattern application to detect matches, followed by exclusion checks based on direct string matching

[2] https://huggingface.co/bvanaken/clinical-assertion-negation-bert

of terms such as “no” or ”not” and the results of the chosen negation detection method. In case of a CD complication match, a context window of up to seven tokens was scanned for uncertainty or exclusion patterns. If the match was not considered negated but linked to B2 or B3 classifications, proximity to mentioning the perianal region was checked for potential phenotype reassignment.

Appendix A.3.3. GPT-4 prompt

Prompt for GPT4-based phenotyping of disease behavior and age at diagnosis. Examples were randomly drawn from the labeled development dataset.

```
Assistant is an expert gastroenterologist good at extracting information from clinical notes.


Below is a clinical note of a Crohn’s disease patient.
Classify the patient according to the Montreal classification (guidelines added) and format output as json.

Inflammatory (NotB2/B3):
- Absence of any prior or current B2 and B3 complication

Stricturing (B2):
Any OR all prior or current of the following:
- Stricture, stenosis or narrowing in any part of colon or small intestine
- Single or multiple luminal narrowings with pre-stenotic dilatation
- Surgical dilation because of Crohn or stricturoplasty
- Small bowel obstruction
- Dilated bowel with transition point or stricture

Penetrating (B3):
Any OR all prior or current of the following:
- Abdominal, abdominal wall, retroperitoneal or pelvic abscess
- Fistula in the small bowel, large bowel, or the enteroenteric, enterocolonic, enterovesicular, enterocutaneous, colocolonic, colovesicular, colovaginal, colocutaneous region
An anastomosis is not enough evidence for B3.
```

```

Perianal Disease (p):
Determined separately from B1/B2/B3 designation if any OR all prior or current:
- Perianal fistula/abscess
- Perirectal or rectal fistula/abscess
- Anal or rectal stricture/stenosis
- Any procedure/surgery for perianal/perirectal/rectal abscess, anal/anorectal dilation or perianal fistula repair (fistulotomy, advancement flap)
- Seton placement
- Any B3 complication identified during rectal examination
Additionally extract the patients’ age at Crohn’s diagnosis in years (no four-digit year number but age), the year of Crohn’s diagnosis or Crohn’s disease duration in years (recalculate months to years by deciving by 12).
Only report the number if you are certain it refers to the Crohn’s disease diagnosis of the patient, no other diseases or family members.

Make sure the answer is correct and don’t output false content. Only extract the values requested.

Here are some examples:

Input: ‘‘‘Crohn’s with history of perianal fistula presenting with recurrent urinary tract symptoms. Assess for rectovaginal or vesicular fistula.‘‘‘
JSON: ‘‘‘
{
"disease_behavior": "Not B2/B3",
"perianal_disease": "1",
"age_at_diagnosis": "Not specified",
"year_of_diagnosis": "Not specified",
"disease_duration": "Not specified"
}
‘‘‘

Input: ‘‘‘Surgeries: Open ileocolic resection, laparatomy due to bowel obstruction.‘‘‘
```

```
JSON: ‘‘‘
{
"disease_behavior": "B2",
"perianal_disease": "0",
"age_at_diagnosis": "Not specified",
"year_of_diagnosis": "Not specified",
"disease_duration": "Not specified"
}
‘‘‘

Input: ‘‘‘HPI: 20 y/o man with fistulizing chrohn’s disease diagnosed 11/2012.‘‘‘
JSON: ‘‘‘
{
"disease_behavior": "B3",
"perianal_disease": "0",
"age_at_diagnosis": "Not specified",
"year_of_diagnosis": "2012",
"disease_duration": "Not specified"
}


Here is the input:
‘‘‘
{note}
‘‘‘
```
